# Crystal structure of taxodione isolated from *Taxodium ascendens* (B.)

**DOI:** 10.1107/S205698901700946X

**Published:** 2017-06-30

**Authors:** Rui-Fang Ke, Shi-Cheng Xu, Ping Song, Shi-Hao Deng, Xin-Hua Ma, Xin-Zhou Yang

**Affiliations:** a School of Pharmaceutical Sciences, South-Central University for Nationalities, Wuhan 430074, People’s Republic of China; b College of Chemistry and Life Science, Qinghai University for Nationalities, Xining 810007, People’s Republic of China

**Keywords:** crystal structure, taxodione, *Taxodium ascendens*

## Abstract

The title compound [systematic name: (4b*S*)-4-hy­droxy-2-isopropyl-4b,8,8-trimethyl-4b,5,6,7,8,8a-hexa­hydro­phenanthrene-3,9-dione], C_20_H_26_O_3_, is an abietane-type diterpene, which was isolated from *Taxodium ascendens* (B.). In the crystal, mol­ecules are linked by weak C—H⋯O hydrogen bonds, forming supra­molecular chains propagating along the [001] direction.

## Abstract 2

In the crystal, the mol­ecules are linked by weak C—H⋯O hydrogen bonds, forming supra­molecular chains propagating along the [001] direction.

## Chemical context   

*Taxodium ascendens* Brongn belongs to the plant family Taxodiaceae and is native to the south-east of North America and can grow up to 25 m in height. It has yellow or orange–yellow seedballs, which mature in October. The plant is widely spread over southern China (*e.g.*, Zhejiang, Henan, Jiangsu, Hubei and Yunnan Provinces) and because of its tolerance of water and drought, it has been used in the landscape at watersides. Previous chemical investigations of extracts isolated from the seeds of *Taxodium ascendens* (B.) revealed the presence of diterpenoids with an abietane framework, including as 6,7-de­hydro­royleanone, salvinolone and xanthoperol (Kusumoto *et al.*, 2009 ▸; González, 2015 ▸). Terpenoids, and in particular diterpenoids, are one of the most important classes of secondary metabolites found in the family Taxodiaceae, and have captured much attention in recent years due to their diverse bioactivities (Burmistrova *et al.*, 2013 ▸; Iwamoto *et al.*, 2001 ▸). In addition, the plant contains lignans and flavonoids (Si *et al.*, 2001 ▸; Otto & Wilde, 2001 ▸) and anti­bacterial and inhibitory activity has been reported (Starks *et al.*, 2014 ▸; Zhang *et al.*, 2009 ▸). A detailed phytochemical investigation of a petroleum extract of the seeds of *Taxodium ascendens* Brongn has been carried out and a series of diterpenoids have been isolated, including the title compound taxodione, that show many biological properties including anti­bacterial (Yang *et al.*, 2001 ▸), anti­oxidant (Kolak *et al.*, 2009 ▸), anti­fungal (Topçu & Gören, 2007 ▸), and anti­cholinesterase activities (Topcu *et al.*, 2013 ▸). Moreover, cytotoxic and tumor inhibitory properties of taxodione have been investigated by *in vivo* experiments (Abou Dahab *et al.*, 2007 ▸). Herein we present the crystal structure of the title compound in order to establish unambiguously the stereochemical features of this natural product. The compound is soluble in chloro­form but has poor solubility in methanol.
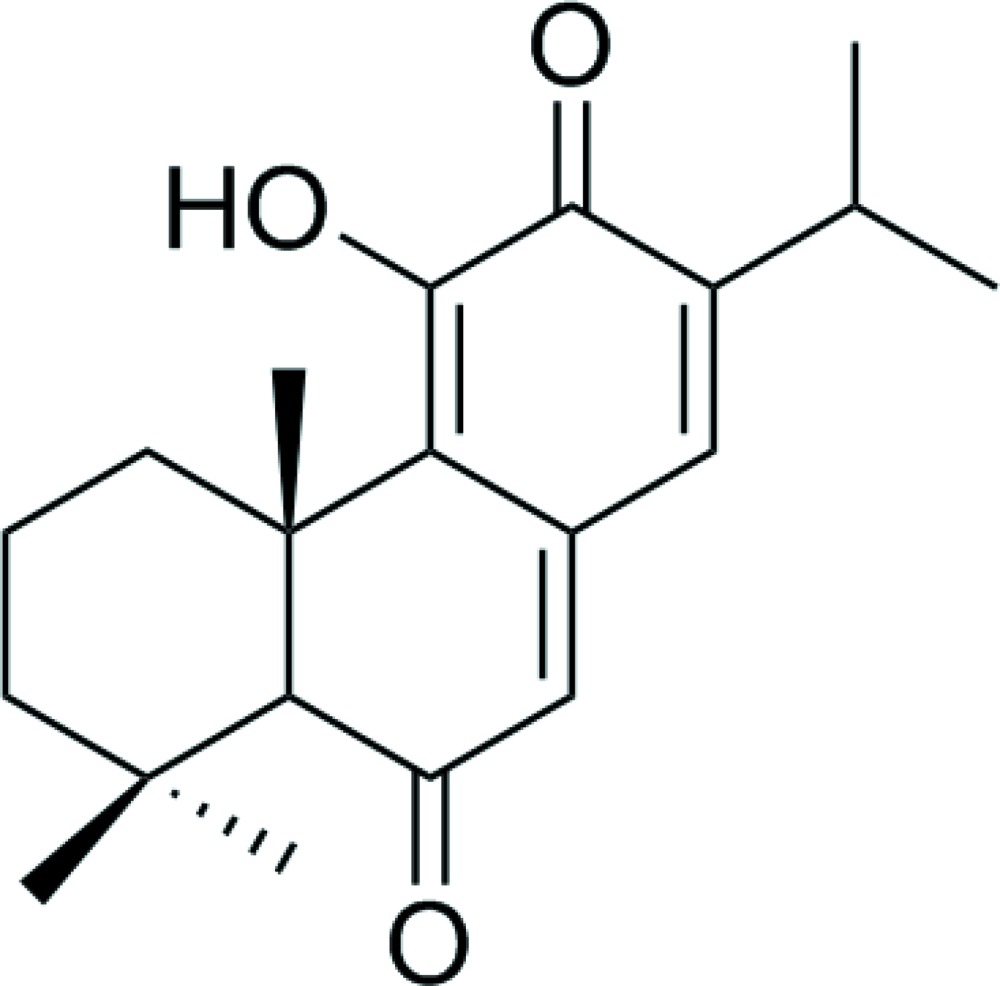


## Structural commentary   

**Figure 1:**
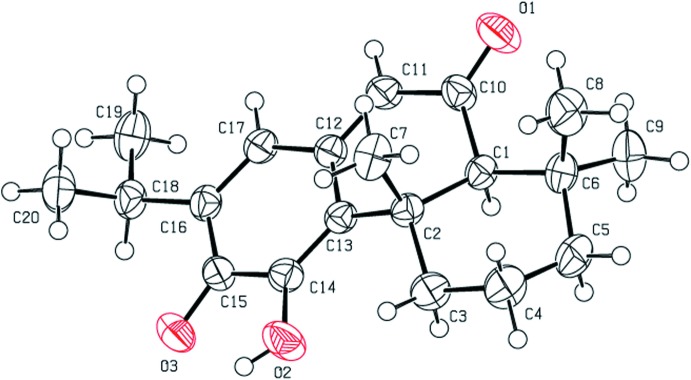
The mol­ecular structure of the title compound, showing 50% probability displacement ellipsoids. A packing diagram of the title compound, with hydrogen bonds shown as dashed lines.

The mol­ecular structure of the title abietane diterpene is shown in Fig. 1 ▸. The structure contains one hydroxyl group located at atom C11, two ketone groups at C6 and C12 and three double bonds located between atoms C7 and C8, C9 and C11, and C13 and C14. An intra­molecular O2—H2⋯O3 hydrogen bond (Fig. 1 ▸) stabilizes the mol­ecular structure. The C14—C13—C12—C11 [−175.83 (19)°], C2—C13—C12—C17 [−168.47 (17)°], C3—C2—C1—C10 [178.98 (16)°] and C13—C2—C1—C6 [−169.12 (16)] torsion angles describe the geometry at the junctions of the three rings.

## Supra­molecular features   

**Figure 2:**
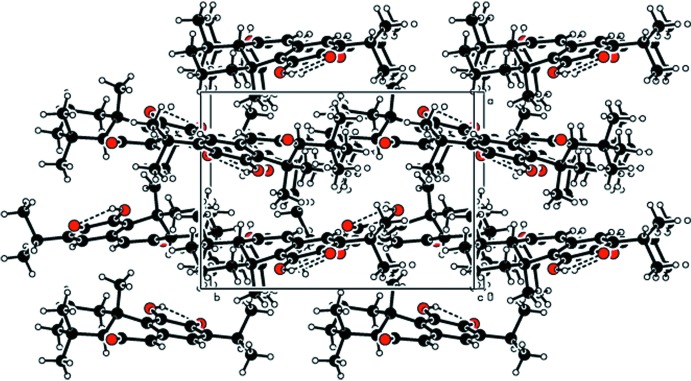
The packing of the title compound.

In the crystal, mol­ecules are linked by weak C—H⋯O hydrogen bonds, forming chains along [001] (Table 1 ▸ and Fig. 2 ▸).

## Database survey   

A search of Cambridge Structural Database found no compounds with a similar structure to the title compound but a series of abietane-type diterpenoids has been reported such as horminone (Xiao *et al.*, 2000 ▸) and 7α,12-di­hydroxy-8,12-abietadiene-11,14-dione [or (4b*S*,8a*S*,10*R*)-3,10-dihy­droxy-2-isopropyl-4b,8,8-trimethyl-1,4,4b,5,6,7,8,8a,9,10-deca­hydro­phenanthrene-1,4-dione] (Razak *et al.*, 2010 ▸).

## Synthesis and crystallization   

Taxodione was isolated from the seeds of *Taxodium ascendens* collected in Wuhan, China, in December 2015 (SC0725). The air-dried seeds of *Taxodium ascendens* (4.6 kg) were extracted with 95% EtOH and then treated with petroleum ether, ethyl acetate and *n*-butyl alcohol to give a PE extract (352 g), EtOAc extract (343 g) and *n*-BuOH extract (372 g). The EtOAc extract (343 g) was subjected to normal-phase silica gel column chromatography (300-400 mesh) with a gradient solvent system of CH_2_Cl_2_–MeOH (1:0–0:1, *v*/*v*, containing 0.1% formic acid) to give fifteen major fractions F1–F15. F5 (13 g) was subjected to sephadex LH-20 CC (CH_2_Cl_2_–MeOH, 3:1, containing 0.1% formic acid) to afford four fractions F5-1–F5-4. F5-2 was purified by semipreparative HPLC (CNCH_3_/H_2_O, 10:90→100:0, 40 min, containing 0.1% formic acid in both phases) to give a yellow solid, which was recrystallized from CH_2_Cl_2_:MeOH (7:1) affording yellow prismatic crystals suitable for X-ray diffraction analysis. For the ^1^H and ^13^C NMR data of taxodione, see Masahiro *et al.* (2010 ▸).

## Refinement   

Crystal data, data collection and structure refinement details are summarized in Table 2 ▸. Hydrogen atoms were positioned with idealized geometry and refined isotropically using a riding model with C—H = 0.97 Å (–CH_3_, allowing for rotation), C—H = 0.98 Å (–CH_2_), C—H = 0.99 Å, (–CH), C–H = 0.94 Å (–CH_2_), and *U*_iso_(H) = 1.5*U*_eq_(CH_3_) and *U*_iso_(H) = 1.2*U*_eq_(CH,CH_2_), with the exception of the O—H hydrogen atom, which was refined freely, but with *U*_iso_(H) = 1.5*U*_eq_(O).

## Supplementary Material

Crystal structure: contains datablock(s) I, global. DOI: 10.1107/S205698901700946X/xu5903sup1.cif

Structure factors: contains datablock(s) I. DOI: 10.1107/S205698901700946X/xu5903Isup2.hkl

Supporting information file. DOI: 10.1107/S205698901700946X/xu5903Isup3.cdx

Supporting information file. DOI: 10.1107/S205698901700946X/xu5903Isup4.cml

CCDC reference: 1551128

Additional supporting information:  crystallographic information; 3D view; checkCIF report

## Figures and Tables

**Table 1:** Hydrogen-bond geometry (Å, °)

*D*—H⋯*A*	*D*—H	H⋯*A*	*D*⋯*A*	*D*—H⋯*A*
O2—H2⋯O3	0.82	2.06	2.554 (2)	118
C11—H11⋯O3^i^	0.93	2.63	3.502 (2)	156

Symmetry code: (i) 
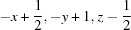
.

**Table 2:** Experimental details

Crystal data	
Chemical formula	C_20_H_26_O_3_
*M*_r_	314.41
Crystal system, space group	Orthorhombic, *P*2_1_2_1_2_1_
Temperature (K)	296
*a*, *b*, *c* (Å)	9.5008 (15), 13.220 (2), 13.584 (2)
*V* (Å^3^)	1706.1 (5)
*Z*	4
Radiation type	Mo *K*α
μ (mm^−1^)	0.08
Crystal size (mm)	0.30 × 0.20 × 0.20

Data collection	
Diffractometer	Bruker APEXII CCD
Absorption correction	–
No. of measured, independent and observed [*I* > 2σ(*I*)] reflections	12903, 3355, 3111
*R*_int_	0.046
(sin θ/λ)_max_ (Å^−1^)	0.617

Refinement	
*R*[*F* ^2^ > 2σ(*F* ^2^)], *wR*(*F* ^2^), *S*	0.034, 0.088, 1.05
No. of reflections	3355
No. of parameters	215
H-atom treatment	H-atom parameters constrained
Δρ_max_, Δρ_min_ (e Å^−3^)	0.17, −0.20

Computer programs: *APEX2* and *SAINT* (Bruker, 2007 ▸), *SHELXS97* and *SHELXTL* (Sheldrick, 2008 ▸) and *SHELXL2014* (Sheldrick, 2015 ▸).

## supplementary crystallographic information

### Crystal data

**Table:**

C_20_H_26_O_3_	*D*_x_ = 1.224 Mg m^−^^3^
*M**_r_* = 314.41	Mo *K*α radiation, λ = 0.71073 Å
Orthorhombic, *P*2_1_2_1_2_1_	Cell parameters from 6770 reflections
*a* = 9.5008 (15) Å	θ = 2.6–30.9°
*b* = 13.220 (2) Å	µ = 0.08 mm^−^^1^
*c* = 13.584 (2) Å	*T* = 296 K
*V* = 1706.1 (5) Å^3^	Prism, yellow
*Z* = 4	0.30 × 0.20 × 0.20 mm
*F*(000) = 680	

### Data collection

**Table:**

Bruker APEXII CCD diffractometer	*R*_int_ = 0.046
φ and ω scans	θ_max_ = 26.0°, θ_min_ = 2.2°
12903 measured reflections	*h* = −11→11
3355 independent reflections	*k* = −16→16
3111 reflections with *I* > 2σ(*I*)	*l* = −16→16

### Refinement

**Table:**

Refinement on *F*^2^	Hydrogen site location: inferred from neighbouring sites
Least-squares matrix: full	H-atom parameters constrained
*R*[*F*^2^ > 2σ(*F*^2^)] = 0.034	*w* = 1/[σ^2^(*F*_o_^2^) + (0.0417*P*)^2^ + 0.2288*P*] where *P* = (*F*_o_^2^ + 2*F*_c_^2^)/3
*wR*(*F*^2^) = 0.088	(Δ/σ)_max_ < 0.001
*S* = 1.05	Δρ_max_ = 0.17 e Å^−^^3^
3355 reflections	Δρ_min_ = −0.20 e Å^−^^3^
215 parameters	Extinction correction: SHELXL2014 (Sheldrick, 2015), Fc^*^=kFc[1+0.001xFc^2^λ^3^/sin(2θ)]^-1/4^
0 restraints	Extinction coefficient: 0.062 (5)

### Special details

**Table:**

Geometry. All esds (except the esd in the dihedral angle between two l.s. planes) are estimated using the full covariance matrix. The cell esds are taken into account individually in the estimation of esds in distances, angles and torsion angles; correlations between esds in cell parameters are only used when they are defined by crystal symmetry. An approximate (isotropic) treatment of cell esds is used for estimating esds involving l.s. planes.

### Fractional atomic coordinates and isotropic or equivalent isotropic displacement parameters (Å^2^)

**Table:**

	*x*	*y*	*z*	*U*_iso_*/*U*_eq_	
O3	0.17963 (19)	0.52283 (12)	1.10546 (10)	0.0539 (4)	
O1	0.2584 (2)	0.83099 (11)	0.64710 (11)	0.0647 (5)	
C5	0.2103 (3)	1.03778 (15)	0.89721 (18)	0.0541 (6)	
H5A	0.2972	1.0319	0.9343	0.065*	
H5B	0.1947	1.1090	0.8841	0.065*	
C4	0.0911 (3)	0.99858 (15)	0.95940 (19)	0.0583 (7)	
H4A	0.0031	1.0060	0.9238	0.070*	
H4B	0.0847	1.0380	1.0194	0.070*	
C3	0.1139 (3)	0.88729 (15)	0.98514 (16)	0.0484 (5)	
H3A	0.0359	0.8639	1.0252	0.058*	
H3B	0.1992	0.8808	1.0238	0.058*	
C2	0.12591 (19)	0.81999 (13)	0.89322 (13)	0.0326 (4)	
C13	0.1741 (2)	0.71188 (13)	0.91885 (13)	0.0311 (4)	
C12	0.2329 (2)	0.65054 (12)	0.83944 (13)	0.0322 (4)	
C17	0.2566 (2)	0.54251 (13)	0.85234 (14)	0.0363 (4)	
H17	0.2872	0.5051	0.7985	0.044*	
C16	0.2365 (2)	0.49443 (13)	0.93772 (13)	0.0349 (4)	
C18	0.2533 (2)	0.38153 (13)	0.95424 (15)	0.0409 (5)	
H18	0.3060	0.3724	1.0156	0.049*	
C19	0.3352 (3)	0.32924 (15)	0.87312 (19)	0.0558 (6)	
H19A	0.4261	0.3603	0.8666	0.084*	
H19B	0.2848	0.3353	0.8121	0.084*	
H19C	0.3465	0.2590	0.8892	0.084*	
C20	0.1092 (3)	0.33257 (16)	0.96823 (19)	0.0541 (6)	
H20A	0.0561	0.3382	0.9084	0.081*	
H20B	0.0599	0.3663	1.0205	0.081*	
H20C	0.1210	0.2625	0.9847	0.081*	
C14	0.1554 (2)	0.66396 (14)	1.00567 (14)	0.0358 (4)	
O2	0.10090 (19)	0.70666 (12)	1.08780 (10)	0.0547 (4)	
H2	0.0925	0.6635	1.1307	0.082*	
C15	0.1912 (2)	0.55593 (14)	1.02095 (14)	0.0369 (4)	
C6	0.2279 (2)	0.98258 (13)	0.79930 (15)	0.0403 (5)	
C8	0.1081 (3)	1.01246 (17)	0.72952 (19)	0.0573 (6)	
H8A	0.1165	0.9751	0.6692	0.086*	
H8B	0.1135	1.0836	0.7158	0.086*	
H8C	0.0193	0.9974	0.7599	0.086*	
C9	0.3670 (3)	1.01768 (17)	0.7533 (2)	0.0574 (6)	
H9A	0.4438	0.9988	0.7954	0.086*	
H9B	0.3657	1.0899	0.7456	0.086*	
H9C	0.3786	0.9864	0.6900	0.086*	
C1	0.2375 (2)	0.86693 (12)	0.82138 (13)	0.0320 (4)	
H1	0.3273	0.8591	0.8561	0.038*	
C10	0.2524 (2)	0.80036 (13)	0.73123 (13)	0.0387 (4)	
C11	0.2643 (2)	0.69172 (13)	0.75146 (13)	0.0389 (4)	
H11	0.2949	0.6493	0.7012	0.047*	
C7	−0.0201 (2)	0.80774 (16)	0.84503 (18)	0.0471 (5)	
H7A	−0.0805	0.7701	0.8882	0.071*	
H7B	−0.0104	0.7723	0.7837	0.071*	
H7C	−0.0602	0.8733	0.8332	0.071*	

### Atomic displacement parameters (Å^2^)

**Table:**

	*U*^11^	*U*^22^	*U*^33^	*U*^12^	*U*^13^	*U*^23^
O3	0.0756 (11)	0.0484 (8)	0.0378 (8)	−0.0038 (8)	0.0044 (8)	0.0116 (7)
O1	0.1165 (15)	0.0410 (7)	0.0366 (7)	0.0117 (10)	0.0151 (10)	0.0065 (6)
C5	0.0723 (16)	0.0303 (9)	0.0596 (13)	−0.0035 (10)	0.0031 (12)	−0.0107 (9)
C4	0.0842 (18)	0.0339 (11)	0.0567 (13)	0.0080 (11)	0.0172 (13)	−0.0137 (10)
C3	0.0671 (15)	0.0355 (10)	0.0427 (11)	0.0047 (10)	0.0112 (11)	−0.0102 (9)
C2	0.0355 (9)	0.0279 (8)	0.0345 (9)	0.0024 (7)	0.0033 (8)	−0.0043 (7)
C13	0.0319 (9)	0.0296 (8)	0.0319 (9)	0.0002 (7)	0.0010 (7)	−0.0032 (7)
C12	0.0353 (9)	0.0287 (8)	0.0325 (8)	0.0020 (7)	0.0003 (7)	−0.0013 (7)
C17	0.0443 (10)	0.0285 (8)	0.0361 (9)	0.0015 (8)	0.0006 (9)	−0.0037 (7)
C16	0.0360 (10)	0.0288 (8)	0.0398 (9)	−0.0019 (7)	−0.0065 (8)	0.0030 (7)
C18	0.0463 (12)	0.0293 (8)	0.0471 (10)	−0.0021 (9)	−0.0104 (10)	0.0079 (8)
C19	0.0617 (14)	0.0276 (9)	0.0780 (16)	0.0029 (9)	0.0017 (12)	−0.0003 (10)
C20	0.0544 (14)	0.0397 (11)	0.0681 (14)	−0.0118 (10)	−0.0038 (11)	0.0094 (11)
C14	0.0364 (10)	0.0369 (9)	0.0341 (9)	−0.0001 (8)	0.0029 (7)	−0.0025 (7)
O2	0.0766 (12)	0.0513 (9)	0.0364 (8)	0.0070 (8)	0.0202 (8)	0.0013 (7)
C15	0.0382 (10)	0.0385 (9)	0.0340 (10)	−0.0063 (8)	−0.0013 (8)	0.0053 (8)
C6	0.0446 (11)	0.0264 (8)	0.0499 (11)	0.0006 (8)	0.0003 (9)	0.0003 (8)
C8	0.0666 (15)	0.0361 (10)	0.0693 (15)	0.0112 (10)	−0.0097 (12)	0.0083 (10)
C9	0.0596 (15)	0.0385 (11)	0.0740 (15)	−0.0057 (10)	0.0103 (13)	0.0092 (11)
C1	0.0332 (9)	0.0260 (8)	0.0367 (9)	0.0030 (7)	−0.0013 (8)	−0.0014 (7)
C10	0.0497 (11)	0.0314 (8)	0.0349 (9)	0.0050 (9)	0.0069 (9)	0.0023 (7)
C11	0.0549 (12)	0.0294 (8)	0.0325 (8)	0.0058 (8)	0.0059 (9)	−0.0046 (7)
C7	0.0344 (10)	0.0387 (10)	0.0683 (14)	0.0030 (8)	−0.0027 (10)	0.0005 (10)

### Geometric parameters (Å, º)

**Table:**

O3—C15	1.233 (2)	C19—H19A	0.9600
O1—C10	1.214 (2)	C19—H19B	0.9600
C5—C4	1.506 (4)	C19—H19C	0.9600
C5—C6	1.526 (3)	C20—H20A	0.9600
C5—H5A	0.9700	C20—H20B	0.9600
C5—H5B	0.9700	C20—H20C	0.9600
C4—C3	1.528 (3)	C14—O2	1.353 (2)
C4—H4A	0.9700	C14—C15	1.483 (3)
C4—H4B	0.9700	O2—H2	0.8200
C3—C2	1.538 (2)	C6—C8	1.533 (3)
C3—H3A	0.9700	C6—C9	1.534 (3)
C3—H3B	0.9700	C6—C1	1.561 (2)
C2—C13	1.541 (2)	C8—H8A	0.9600
C2—C7	1.542 (3)	C8—H8B	0.9600
C2—C1	1.569 (3)	C8—H8C	0.9600
C13—C14	1.350 (3)	C9—H9A	0.9600
C13—C12	1.461 (2)	C9—H9B	0.9600
C12—C11	1.347 (2)	C9—H9C	0.9600
C12—C17	1.456 (2)	C1—C10	1.515 (2)
C17—C16	1.336 (3)	C1—H1	0.9800
C17—H17	0.9300	C10—C11	1.467 (2)
C16—C15	1.458 (3)	C11—H11	0.9300
C16—C18	1.518 (2)	C7—H7A	0.9600
C18—C19	1.515 (3)	C7—H7B	0.9600
C18—C20	1.527 (3)	C7—H7C	0.9600
C18—H18	0.9800		
			
C4—C5—C6	114.00 (18)	C18—C20—H20B	109.5
C4—C5—H5A	108.8	H20A—C20—H20B	109.5
C6—C5—H5A	108.8	C18—C20—H20C	109.5
C4—C5—H5B	108.8	H20A—C20—H20C	109.5
C6—C5—H5B	108.8	H20B—C20—H20C	109.5
H5A—C5—H5B	107.6	C13—C14—O2	125.06 (17)
C5—C4—C3	110.7 (2)	C13—C14—C15	122.97 (17)
C5—C4—H4A	109.5	O2—C14—C15	111.96 (16)
C3—C4—H4A	109.5	C14—O2—H2	109.5
C5—C4—H4B	109.5	O3—C15—C16	123.39 (18)
C3—C4—H4B	109.5	O3—C15—C14	116.86 (18)
H4A—C4—H4B	108.1	C16—C15—C14	119.75 (16)
C4—C3—C2	112.45 (18)	C5—C6—C8	109.54 (18)
C4—C3—H3A	109.1	C5—C6—C9	107.74 (18)
C2—C3—H3A	109.1	C8—C6—C9	108.04 (18)
C4—C3—H3B	109.1	C5—C6—C1	107.90 (16)
C2—C3—H3B	109.1	C8—C6—C1	114.51 (16)
H3A—C3—H3B	107.8	C9—C6—C1	108.91 (16)
C3—C2—C13	112.04 (15)	C6—C8—H8A	109.5
C3—C2—C7	109.80 (17)	C6—C8—H8B	109.5
C13—C2—C7	105.41 (15)	H8A—C8—H8B	109.5
C3—C2—C1	109.04 (15)	C6—C8—H8C	109.5
C13—C2—C1	107.86 (14)	H8A—C8—H8C	109.5
C7—C2—C1	112.67 (15)	H8B—C8—H8C	109.5
C14—C13—C12	115.76 (15)	C6—C9—H9A	109.5
C14—C13—C2	126.42 (16)	C6—C9—H9B	109.5
C12—C13—C2	117.50 (15)	H9A—C9—H9B	109.5
C11—C12—C17	117.98 (16)	C6—C9—H9C	109.5
C11—C12—C13	121.04 (15)	H9A—C9—H9C	109.5
C17—C12—C13	120.97 (15)	H9B—C9—H9C	109.5
C16—C17—C12	123.29 (17)	C10—C1—C6	114.79 (15)
C16—C17—H17	118.4	C10—C1—C2	109.64 (14)
C12—C17—H17	118.4	C6—C1—C2	117.86 (15)
C17—C16—C15	116.75 (16)	C10—C1—H1	104.3
C17—C16—C18	125.52 (17)	C6—C1—H1	104.3
C15—C16—C18	117.70 (16)	C2—C1—H1	104.3
C19—C18—C16	113.28 (17)	O1—C10—C11	119.97 (17)
C19—C18—C20	110.95 (18)	O1—C10—C1	124.87 (17)
C16—C18—C20	109.92 (17)	C11—C10—C1	115.13 (15)
C19—C18—H18	107.5	C12—C11—C10	123.03 (16)
C16—C18—H18	107.5	C12—C11—H11	118.5
C20—C18—H18	107.5	C10—C11—H11	118.5
C18—C19—H19A	109.5	C2—C7—H7A	109.5
C18—C19—H19B	109.5	C2—C7—H7B	109.5
H19A—C19—H19B	109.5	H7A—C7—H7B	109.5
C18—C19—H19C	109.5	C2—C7—H7C	109.5
H19A—C19—H19C	109.5	H7A—C7—H7C	109.5
H19B—C19—H19C	109.5	H7B—C7—H7C	109.5
C18—C20—H20A	109.5		
			
C6—C5—C4—C3	60.6 (3)	C17—C16—C15—C14	6.4 (3)
C5—C4—C3—C2	−58.9 (3)	C18—C16—C15—C14	−172.15 (17)
C4—C3—C2—C13	170.16 (19)	C13—C14—C15—O3	174.55 (19)
C4—C3—C2—C7	−73.1 (2)	O2—C14—C15—O3	−6.4 (3)
C4—C3—C2—C1	50.8 (2)	C13—C14—C15—C16	−5.8 (3)
C3—C2—C13—C14	26.0 (3)	O2—C14—C15—C16	173.20 (18)
C7—C2—C13—C14	−93.4 (2)	C4—C5—C6—C8	72.5 (2)
C1—C2—C13—C14	146.02 (18)	C4—C5—C6—C9	−170.2 (2)
C3—C2—C13—C12	−160.72 (17)	C4—C5—C6—C1	−52.8 (3)
C7—C2—C13—C12	79.9 (2)	C5—C6—C1—C10	178.84 (18)
C1—C2—C13—C12	−40.7 (2)	C8—C6—C1—C10	56.6 (2)
C14—C13—C12—C11	−175.83 (19)	C9—C6—C1—C10	−64.5 (2)
C2—C13—C12—C11	10.2 (3)	C5—C6—C1—C2	47.3 (2)
C14—C13—C12—C17	5.5 (3)	C8—C6—C1—C2	−74.9 (2)
C2—C13—C12—C17	−168.47 (17)	C9—C6—C1—C2	164.01 (18)
C11—C12—C17—C16	176.3 (2)	C3—C2—C1—C10	178.98 (16)
C13—C12—C17—C16	−5.0 (3)	C13—C2—C1—C10	57.08 (18)
C12—C17—C16—C15	−1.2 (3)	C7—C2—C1—C10	−58.84 (19)
C12—C17—C16—C18	177.20 (19)	C3—C2—C1—C6	−47.2 (2)
C17—C16—C18—C19	16.5 (3)	C13—C2—C1—C6	−169.12 (16)
C15—C16—C18—C19	−165.18 (19)	C7—C2—C1—C6	75.0 (2)
C17—C16—C18—C20	−108.3 (2)	C6—C1—C10—O1	0.8 (3)
C15—C16—C18—C20	70.1 (2)	C2—C1—C10—O1	136.1 (2)
C12—C13—C14—O2	−179.14 (19)	C6—C1—C10—C11	178.87 (18)
C2—C13—C14—O2	−5.7 (3)	C2—C1—C10—C11	−45.8 (2)
C12—C13—C14—C15	−0.3 (3)	C17—C12—C11—C10	−176.54 (19)
C2—C13—C14—C15	173.14 (18)	C13—C12—C11—C10	4.8 (3)
C17—C16—C15—O3	−174.0 (2)	O1—C10—C11—C12	−167.3 (2)
C18—C16—C15—O3	7.5 (3)	C1—C10—C11—C12	14.5 (3)

### Hydrogen-bond geometry (Å, º)

**Table:**

*D*—H···*A*	*D*—H	H···*A*	*D*···*A*	*D*—H···*A*
O2—H2···O3	0.82	2.06	2.554 (2)	118
C11—H11···O3^i^	0.93	2.63	3.502 (2)	156

Symmetry code: (i) −*x*+1/2, −*y*+1, *z*−1/2.

## References

[bb1] Abou Dahab, M. A., El-Bahr, M. K., Taha, H. S., Habib, A. M., Bekheet, S. A., Gabr, A. M. M. & Refaat, A. (2007). *J. Appl. Sci. Res.* **3**, 1987–1996.

[bb2] Bruker (2007). *APEX2* and *SAINT*. Bruker AXS Inc., Madison, Wisconsin, USA.

[bb3] Burmistrova, O., Simões, M. F., Rijo, P., Quintana, J., Bermejo, J. & Estévez, F. (2013). *J. Nat. Prod.* **76**, 1413–1423. 10.1021/np400172k 23865778

[bb4] González, M. A. (2015). *Nat. Prod. Rep.* **32**, 684–704. 10.1039/c4np00110a 25643290

[bb5] Iwamoto, M., Ohtsu, H., Tokuda, H., Nishino, H., Matsunaga, S. & Tanaka, R. (2001). *Bioorg. Med. Chem.* **9**, 1911–1921. 10.1016/s0968-0896(01)00099-2 11425594

[bb6] Kolak, U., Kabouche, A., Öztürk, M., Kabouche, Z., Topçu, G. & Ulubelen, A. (2009). *Phytochem. Anal.* **20**, 320–327. 10.1002/pca.1130 19402189

[bb7] Kusumoto, N., Ashitani, T., Hayasaka, Y., Murayama, T., Ogiyama, K. & Takahashi, K. (2009). *J. Chem. Ecol.* **35**, 635–642. 10.1007/s10886-009-9646-0 19475449

[bb8] Masahiro, T., Jun, K., Takashi, Y., Tomoyuki, O. & Yusuke, M. (2010). *Chem. Pharm. Bull.* **58**, 818–824.

[bb9] Otto, A. & Wilde, V. (2001). *Bot. Rev.* **67**, 141–238.

[bb10] Razak, I. A., Salae, A. W., Chantrapromma, S., Karalai, C. & Fun, H.-K. (2010). *Acta Cryst.* E**66**, o1566–o1567. 10.1107/S1600536810020544 PMC3006754 21587809

[bb11] Sheldrick, G. M. (2008). *Acta Cryst.* A**64**, 112–122. 10.1107/S0108767307043930 18156677

[bb12] Sheldrick, G. M. (2015). *Acta Cryst.* C**71**, 3–8.

[bb13] Si, Y., Zhang, C.-K., Yao, X.-H. & Tu, Z.-B. (2001). *J. Wuhan Bot. Res*, **19**, 517–520.

[bb14] Starks, C. M., Norman, V. L., Williams, R. B., Goering, M. G., Rice, S. M., O’Neil-Johnson, M. & Eldridge, G. R. (2014). *Nat. Prod. Commun.* **9**, 1129–1130. 25233589

[bb15] Topçu, G. & Gören, A. C. (2007). *Rec. Nat. Prod.* **1**, 1–16.

[bb16] Topcu, G., Kolak, U., Ozturk, M., Boga, M., Hatipoglu, S. D., Bahadori, F., Culhaoglu, B. & Dirmenci, T. (2013). *Nat. Prod. J*, **3**, 3–9.

[bb17] Xiao, C., Ren, A.-L., Lin, H.-W., Qing, L.-X. & Feng, J.-D. (2000). *Chin. J. Struct. Chem.* **19**, 122–125.

[bb18] Yang, Z., Kitano, Y., Chiba, K., Shibata, N., Kurokawa, H., Doi, Y., Arakawa, Y. & Tada, M. (2001). *Bioorg. Med. Chem.* **9**, 347–356. 10.1016/s0968-0896(00)00253-4 11249127

[bb19] Zhang, Y. M., Tan, N. H., Zeng, G. Z., Adebayo, A. H. & Ji, C. J. (2009). *Fitoterapia*, **80**, 361–363. 10.1016/j.fitote.2009.05.001 19433133

